# Work-Related Musculoskeletal Complaints Among Male Retail Butchers: Multisite Pain, Upper-Extremity Disability and Burnout

**DOI:** 10.3390/healthcare14213789

**Published:** 2026-11-06

**Authors:** Ömer Şevgin, Esra Nur Demir, Ravza Hilal Gümüşçü, Birsu Başak Bulut, Enhar Kılıç, Suzan Aydın

**Affiliations:** 1 Department of Physiotherapy and Rehabilitation, Faculty of Health Sciences, Üsküdar University, Istanbul 34662, Türkiye; esranur.demir@st.uskudar.edu.tr (E.N.D.); ravzahilal.gumuscu@st.uskudar.edu.tr (R.H.G.); birsubasak.bulut@st.uskudar.edu.tr (B.B.B.); enhar.kilic@st.uskudar.edu.tr (E.K.); 2 Department of Physiotherapy and Rehabilitation, Faculty of Health Sciences, İstanbul Gelişim University, Istanbul 34310, Türkiye; suaydin@gelisim.edu.tr

**Keywords:** butchers, musculoskeletal diseases, occupational health, multisite pain, burnout, professional, upper extremity, cross-sectional studies

## Abstract

**Background/Objectives:** Butchering combines repetitive forceful knife work, sustained standing and heavy manual handling, yet data on musculoskeletal complaints in this trade are scarce. We examined the seven-day prevalence of musculoskeletal pain in male retail butchers and the factors associated with multisite pain and upper-extremity disability. **Methods:** In this cross-sectional study, 99 of 112 male butchers affiliated with the Istanbul Chamber of Retail Butchers (participation 88.4%) completed a sociodemographic form, the Standardised Nordic Musculoskeletal Questionnaire (seven-day recall), the QuickDASH and the Maslach Burnout Inventory in face-to-face interviews. Multisite pain was defined a priori as pain in three or more of nine regions. Given marked group imbalance, it was modelled with Firth penalised logistic and Poisson regression; QuickDASH with linear regression. **Results:** Mean age was 38.69 ± 7.53 years; mean experience 16.10 ± 7.36 years. Pain was most frequent in the upper back (66.7%), shoulders (63.6%) and hand/wrist (59.6%); 88.9% (95% CI 81.2–93.7) reported three or more painful regions. Age was the only variable independently associated with multisite pain (OR 1.17, 95% CI 1.05–1.30) and with region count (IRR 1.022, 95% CI 1.010–1.036). No statistically significant association was detected between any burnout dimension and any outcome, but the confidence intervals were wide (e.g., emotional exhaustion, OR 0.73 per SD, 95% CI 0.39–1.39) and small to moderate effects cannot be excluded. **Conclusions:** Multisite pain burden was very high and increased with age, which in this sample cannot be separated from cumulative occupational exposure. The findings highlight the potential importance of physical and ergonomic exposures; because only burnout was measured, no conclusion can be drawn about psychosocial factors more broadly. Findings are exploratory.

## 1. Introduction

Work-related musculoskeletal disorders (WRMSDs) are among the most common occupational health problems worldwide and account for a substantial share of compensation costs, sickness absence and early exit from working life [1]. They arise when soft tissues—muscles, tendons, ligaments, nerves and intervertebral discs—are loaded repetitively, forcefully or in sustained non-neutral postures, and their multifactorial aetiology makes them difficult to attribute to any single exposure [1]. Their occurrence is strongly patterned by occupation rather than evenly distributed: in manually demanding and repetitive trades, point-prevalence estimates for symptoms in at least one body region commonly reach or exceed half of the workforce, and symptom burden tracks the specific physical demands of the job rather than general population risk factors [2]. It is this concentration of risk in particular trades that makes occupation-specific estimates, rather than general population figures, the useful unit for prevention planning.

Butchering is a trade in which several of these exposures occur simultaneously. Carcass handling and meat cutting require repeated forceful knife work with a pinched or power grip, frequent shoulder elevation, trunk flexion and rotation, sustained standing, and manual handling of heavy loads, often in cold and wet environments [3,4]. The presumed pathomechanics follow directly from this profile. Sustained and repeated gripping with the wrist deviated loads the finger flexor tendons and raises pressure within the carpal tunnel; repeated and sustained shoulder elevation above mid-range narrows the subacromial space and loads the rotator cuff tendons under reduced perfusion; and prolonged trunk flexion maintains static erector spinae activity while increasing compressive loading of the lumbar motion segments. In each case the proposed mechanism is cumulative micro-trauma without adequate recovery rather than a single injurious event, which is why symptom burden in this sector rises with exposure duration rather than appearing abruptly [1,4]. Consistent with this account, epidemiological and surveillance work in meat processing has documented a high burden of repetitive-strain and musculoskeletal diagnoses among slaughterers, butchers and deboners [3], narrative and intervention reviews of abattoir and poultry-processing work reach the same conclusion [4,5], and cross-sectional studies in slaughterhouses have reported musculoskeletal pain in the large majority of workers, with the upper limb most frequently affected and workplace risk factors graded by force and repetition [6,7,8].

Research specific to butchers remains limited and heterogeneous. A recent scoping review identified a small body of work reporting twelve-month prevalence estimates ranging from roughly 10% to 88%, most often involving the low back, shoulders, wrists and neck, and concluded that the evidence base is dominated by single-site prevalence surveys with little methodological consistency [9]. The studies closest to the present one differ from it in three respects. First, they have almost all been conducted in industrial slaughterhouses and poultry-processing plants, where tasks are highly subdivided and machine-paced [3,6,7,8], whereas retail butchers in small independent shops perform a broader mix of tasks with less mechanisation and little formal ergonomic oversight. Second, they report region-by-region prevalence, treating each body site as an independent outcome—an approach that the widely used Standardised Nordic Musculoskeletal Questionnaire naturally encourages [10]—whereas pain in several regions simultaneously is more common than single-site pain, is more disabling, and predicts functional limitation and work disability more strongly than pain at any one site [11]. Third, they have assessed either physical symptoms or psychosocial strain, rarely both in the same sample. Measuring upper-limb functional limitation and occupational burnout alongside the distribution of pain in one cohort is useful precisely because it allows the relative contribution of the two domains to be compared within a single exposure setting, rather than inferred across studies with different populations and instruments. In Türkiye specifically, published work on retail butchers is almost absent, although the counting-of-regions approach has been applied productively in other Turkish occupational groups [12].

Alongside physical exposure, psychosocial factors have been proposed as contributors to musculoskeletal symptom burden [13]. Burnout—conceptualised by Maslach and Jackson as emotional exhaustion, depersonalisation and reduced personal accomplishment [14]—is of particular interest because it is by definition work-related, which distinguishes it from generalised anxiety or depressive symptoms, and because the job demands–resources framework provides an explicit mechanism linking sustained demands to energetic depletion and subsequent health complaints [13,15]. The three dimensions are not expected to behave identically with respect to multisite pain. Emotional exhaustion, the energetic component, is the dimension most consistently linked to somatic complaints and to the number of painful regions, plausibly through impaired recovery and sleep [13]; depersonalisation, an interpersonal distancing response, has shown weaker and less consistent associations; and reduced personal accomplishment, an evaluative component, has been related to pain mainly in occupations where professional efficacy is central to the work role [15]. Longitudinal evidence in healthcare workers has linked burnout and occupational stress to multisite pain [13], and occupational stress has been associated with musculoskeletal symptoms in physically demanding sectors such as mining [16]. However, the evidence is not uniform: a recent Turkish cross-sectional study of academicians found no association between overall burnout scores and the number of painful regions, while other psychosocial variables were related to multisite pain [17]; and most supportive findings come from occupations in which emotional labour, rather than physical loading, predominates [13,15]. Whether burnout contributes measurably to musculoskeletal burden in a physically heavy manual trade is therefore an open question, and organisational as well as individual factors may matter in this setting [18].

The practical value of occupation-specific prevalence data lies in what can be done with it, and the preventive and rehabilitative options for work-related musculoskeletal complaints are reasonably well characterised. Interventions in physically demanding sectors typically combine ergonomic modification of the workstation and hand tools with an active component—therapeutic exercise directed at the loaded tissues—and with structured health education covering posture, load handling, work pacing and recovery; reviews of workplace programmes in abattoir and poultry-processing settings indicate that such multicomponent approaches outperform single-element measures and that their effectiveness depends on matching the intervention to the specific task exposures of the setting [4,5]. The best-studied example of the combined educational and exercise-based approach is the back school, in which group education on spinal loading and self-management is delivered alongside supervised strengthening and flexibility training: a systematic review and meta-analysis found clinically relevant reductions in both pain and disability in non-specific back pain [19], and a subsequent randomised controlled trial of an eight-week back school-based programme reported improvements in pain, disability, kinesiophobia and the physical component of quality of life relative to usual activity [20]. Programmes of this kind are generally delivered multidisciplinarily, involving physiotherapists and occupational health practitioners and, where the workplace permits, ergonomists and employers. Whether any of this transfers to retail butchers is unknown, however, because the descriptive groundwork that intervention design requires—which regions are affected, how widely pain is distributed across the body, and which worker characteristics identify those most affected—has not been established for this trade. Characterising that burden is a precondition for designing an intervention rather than importing one.

The present study was conducted to address these gaps. Its aims were, first, to describe the seven-day prevalence and distribution of musculoskeletal pain among male retail butchers in Istanbul and to identify the individual and occupational factors associated with multisite pain; and second, to examine which factors are associated with upper-extremity disability as measured by the QuickDASH. To our knowledge this is the first study to characterise multisite musculoskeletal pain in Turkish retail butchers, and the first in any butcher population to measure pain distribution, upper-limb functional limitation and occupational burnout together. We tested three hypotheses: (H1) multisite pain, defined as pain in three or more body regions, would be highly prevalent in this group; (H2) age and occupational experience would be positively associated with the number of painful regions; and (H3) higher burnout, and in particular emotional exhaustion, would be associated with a greater number of painful regions and with greater upper-extremity disability.

## 2. Materials and Methods

### 2.1. Study Design and Setting

This was a single-centre, cross-sectional study of male retail butchers affiliated with the Istanbul Chamber of Retail Butchers (İstanbul Perakendeci Kasaplar Esnaf Odası), conducted between July and September 2026, after ethical approval had been obtained. The study is reported in accordance with the STROBE statement for cross-sectional studies.

Eligible members were informed about the study through the Chamber and at their workplaces. All questionnaires were administered in face-to-face interviews by members of the research team, who had been trained by the supervising investigator (Ö.Ş.) before data collection began. Several measures were taken to limit social desirability and interviewer bias. Interviewers worked from a fixed script and read every item and response option verbatim, in the order printed on the form, without paraphrasing, prompting or commenting on answers; this also minimised missing and uninterpretable responses. Interviews were conducted in a private area of the shop, out of hearing of employers, colleagues and customers. Participants were told explicitly, before the first item, that there were no right or wrong answers, that their responses would be anonymised at data entry, and that neither the Chamber nor their employer would receive individual data, so that their answers could have no bearing on their employment or Chamber membership. No incentive was offered. Because the interviewers were members of the research team rather than independent assessors, residual social desirability bias cannot be excluded, and this is addressed in the Limitations. Every participant received a verbal and written explanation of the study and provided written informed consent before completing any instrument.

### 2.2. Participants

Inclusion criteria were active membership of the Istanbul Chamber of Retail Butchers and current work as a butcher; aged between 25 and 50 years; at least five years of continuous active work in the trade; and the ability to read and write Turkish. The single exclusion criterion was concurrent employment in a second occupation, which was applied so that reported symptoms could be attributed to butchery rather than to mixed occupational exposure.

Of 112 butchers approached, 99 agreed to participate and completed all instruments, giving a participation rate of 88.4%. All 13 non-participants declined without giving a reason, and reasons for refusal were not systematically recorded during recruitment; we therefore cannot characterise the non-participants or compare them with those who took part, which is acknowledged in the Limitations. There were no missing data among participants. All participants were men; no female butchers were identified within the sampling frame during the recruitment period. No a priori sample size calculation was performed. The study was conducted as a student research project with a fixed recruitment window and aimed to enrol the entire eligible membership of a single chamber rather than a statistically derived target; this is acknowledged as a limitation. The achieved sample nonetheless supports reasonable precision for the primary descriptive aim: with *n* = 99, a prevalence estimate near 89% is bounded by a 95% confidence interval approximately 12 percentage points wide. Precision for the group comparisons is considerably lower. With 11 participants in the 0–2 region group and 88 in the multisite pain group, the design has 80% power to detect only a large standardised difference (Cohen’s d ≈ 0.90) at a two-sided alpha of 0.05, and roughly 50% power at d ≈ 0.63. Comparisons involving the smaller group, including all burnout analyses, should therefore be read as having limited sensitivity to small and moderate effects.

### 2.3. Measures

**Sociodemographic and occupational form.** A purpose-designed form recorded age, marital status, educational attainment, height, weight, years of experience in the trade, daily working hours and weekly working days. Body mass index (BMI) was calculated as weight in kilograms divided by height in metres squared.

**Standardised Nordic Musculoskeletal Questionnaire (NMQ).** The NMQ is a validated instrument for screening musculoskeletal symptoms across nine body regions (neck, shoulders, upper back, elbows, wrists/hands, low back, hips/thighs, knees and ankles/feet) [10]; the Turkish version used here was cross-culturally adapted and validated by Kahraman et al., who reported acceptable internal consistency and test–retest reliability [21]. Only the seven-day recall item was administered. Participants were asked whether they had experienced any trouble (ache, pain or discomfort) in each region at any time during the preceding seven days. All prevalence figures reported here are therefore seven-day point prevalences and are not comparable with the twelve-month period prevalences more commonly reported in the occupational literature [6]. For each region, responses were coded as pain present or absent, and the number of painful regions was summed to produce a count ranging from 0 to 9. Multisite pain was defined a priori as pain in three or more regions. This threshold follows the operational definition established by Kamaleri and colleagues, who assessed pain sites over a seven-day window in the general population and showed that functional consequences rise steeply once three or more sites are affected [11]; the same threshold has been applied in Turkish occupational samples [12,17].

**Pain frequency and intensity.** Participants rated how often they experienced work-related pain on a four-point scale (never, occasionally, often, very often) and rated its usual intensity on a 0–10 visual analogue scale (VAS) anchored at 0 (no pain) and 10 (worst pain imaginable). VAS scores were recorded to the nearest whole number, and the observed range in this sample was 0–8.

**QuickDASH.** The QuickDASH is an 11-item short form of the Disabilities of the Arm, Shoulder and Hand questionnaire, scored from 0 (no disability) to 100 (most severe disability). It was developed as a shortened alternative with measurement properties comparable to the full-length instrument [22]. The Turkish version was adapted and validated by Koldaş Doğan et al., who reported acceptable internal consistency and test–retest reliability [23]. Only the main module was used; the optional work and sports/performing arts modules were not administered. Because the analysis dataset contained scale totals rather than item-level responses, Cronbach’s alpha could not be computed for the present sample; this is stated again in the Limitations.

**Maslach Burnout Inventory (MBI).** Burnout was assessed with the 22-item MBI [14], which yields three subscale scores: emotional exhaustion (9 items), depersonalisation (5 items) and personal accomplishment (8 items). Items are rated on a five-point frequency scale from 0 (never) to 4 (always). The validated Turkish adaptation by Ergin was used [24]. The personal accomplishment items are worded in the direction of achievement and were therefore reverse-scored, so that the subscale analysed here is oriented as reduced personal accomplishment: higher scores indicate a greater sense of diminished professional efficacy, and thus greater burnout, in the same direction as the other two subscales. The three subscales were analysed separately throughout. No composite total score was computed, because summing a reverse-keyed subscale with two directly keyed subscales does not yield a validated composite [17]. As for the QuickDASH, the analysis dataset contained subscale totals rather than item-level responses, so internal consistency for the present sample could not be calculated; the reliability evidence cited is that of the Turkish validation study.

### 2.4. Statistical Analysis

Analyses were performed in IBM SPSS Statistics version 25.0 (IBM Corp., Armonk, NY, USA) and cross-checked in Python 3.11 (Python Software Foundation, Wilmington, DE, USA) using pandas, statsmodels and SciPy; the Firth penalised and Poisson models were fitted in Python. Continuous variables are summarised as mean ± standard deviation with range, and categorical variables as frequency and percentage.

Distributional assumptions were assessed with the Shapiro–Wilk test together with skewness and kurtosis. Because the two pain groups were markedly unequal in size (11 versus 88) and several variables departed from normality within the smaller group, the Mann–Whitney U test was specified as the primary test for between-group comparisons, with the Welch t-test reported alongside it for transparency. Effect sizes are reported as Cohen’s d. Associations between continuous variables were quantified with Spearman’s rank correlation coefficient.

Comparisons of the four scale scores (the three MBI subscales and the QuickDASH) across the nine body regions generated 36 tests. Because this number of comparisons materially inflates the error rate, two complementary corrections were applied to this family of 36 *p*-values. The Holm step-down procedure, which controls the family-wise error rate, was pre-specified as the primary correction, because the regional analyses were exploratory and the cost of a false positive claim about a specific body region was judged greater than the cost of a missed signal. The Benjamini–Hochberg procedure, which controls the false discovery rate and is less conservative, is reported alongside it so that readers can see how sensitive the conclusions are to the choice of criterion. Both are presented in full in the Results, and regional findings are interpreted only if they survive the primary (Holm) correction.

Two sets of regression models were fitted. For the first aim, multisite pain (≥3 regions) was modelled with Firth penalised logistic regression [25] rather than conventional maximum-likelihood logistic regression, because the small number of participants in the reference group yields few events per variable and unpenalised estimates are unstable under such conditions [12]. Penalisation reduces small-sample bias but does not recover the information that a reference group of 11 does not contain, so estimates are interpreted together with their confidence intervals throughout. Odds ratios are reported both per unit and, for the burnout subscales, per standard deviation, because per-unit odds ratios on a 0–36 or 0–20 scale can appear deceptively precise. In parallel, the number of painful regions was modelled directly as a count using Poisson regression, which avoids dichotomising the outcome and uses the full information in the region count. The Poisson assumption of no over-dispersion was checked with both the Pearson χ^2^- and deviance-to-degrees-of-freedom ratios, and model fit was assessed with the corresponding goodness-of-fit tests against the χ^2^ distribution. Age and years of experience were strongly collinear (r = 0.887; variance inflation factor 4.7) and were therefore not entered simultaneously; age was retained in the multivariable models and experience examined univariably. The QuickDASH was deliberately excluded from the predictor set for this aim, because one of its items enquires directly about arm, shoulder or hand pain and therefore overlaps with the outcome being modelled.

For the second aim, QuickDASH score was treated as a dependent variable in a multiple linear regression including the number of painful regions, pain intensity, age, BMI and the three burnout subscales. Model assumptions were examined in full: multicollinearity with variance inflation factors; normality of residuals with the Shapiro–Wilk and Jarque–Bera tests; homoscedasticity with the Breusch–Pagan and White tests; independence of residuals with the Durbin–Watson statistic; and influential observations with Cook’s distance and externally studentised residuals. For consistency of reporting, regression coefficients, odds ratios and standardised mean differences are given to two decimal places with their confidence intervals to the same precision; incidence rate ratios and standardised regression coefficients, whose informative variation lies in the third decimal place, are given to three; and all *p*-values, including multiplicity-adjusted values, are given to three decimal places, with values below 0.001 reported as *p* < 0.001.

Two-sided *p*-values below 0.05 were considered statistically significant. Given the sample size and the number of models fitted, estimates are presented with 95% confidence intervals and interpreted in terms of precision rather than significance alone.

### 2.5. Ethics

The study protocol was approved by the Non-Interventional (Non-Clinical) Research Ethics Committee of Üsküdar University (meeting no. 12, 26 November 2025; approval letter dated 28 November 2025, no. 61351342/020-1612) and was conducted in accordance with the principles of the Declaration of Helsinki. Written informed consent for participation was obtained from every participant prior to data collection.

## 3. Results

### 3.1. Participant Characteristics

The 99 participants had a mean age of 38.69 ± 7.53 years (range 25–50) and a mean of 16.10 ± 7.36 years (range 5–32) of experience in the trade. Mean BMI was 27.09 ± 3.09 kg/m^2^. Participants worked 9.05 ± 0.50 h per day and 5.91 ± 0.35 days per week; 74.8% worked nine hours daily and 92.9% worked six days per week. Three quarters (74.8%) were married, 18.2% single and 7.1% divorced. Educational attainment was secondary school in 22.2%, high school in 22.2% and university in 55.6%. Full descriptive data are presented in Table 1.

### 3.2. Prevalence and Distribution of Musculoskeletal Pain

**Figure 1:**
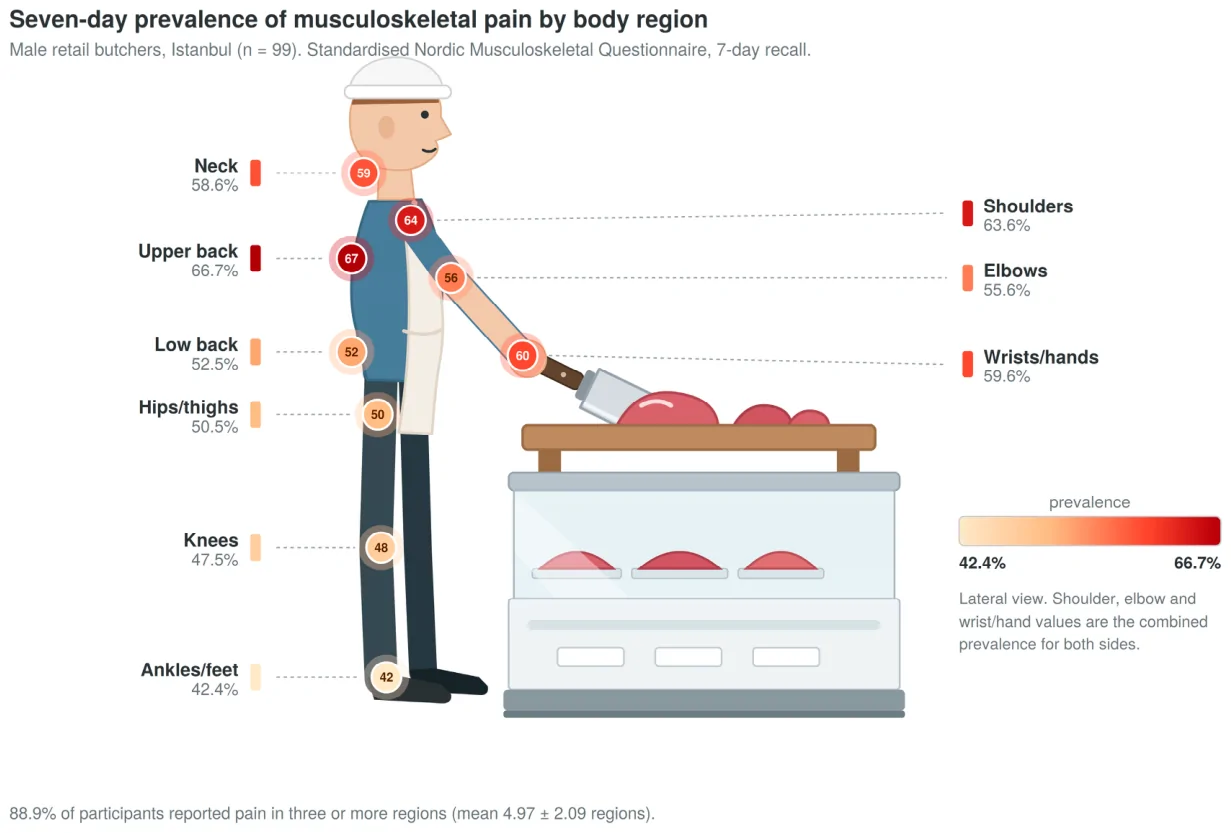
Seven-day prevalence of musculoskeletal pain by body region among male retail butchers (*n* = 99), shown on a lateral body map. Marker colour and the value inside each marker indicate the percentage of participants reporting pain in that region during the preceding seven days. For the shoulders, elbows and wrists/hands the value is the combined prevalence for both sides.

Pain during the preceding seven days was reported most frequently in the upper back (66.7%), followed by the shoulders (63.6%), hand/wrist (59.6%), neck (58.6%), elbows (55.6%), low back (52.5%), hip/thigh (50.5%), knees (47.5%) and ankle/foot (42.4%) (Table 2; Figure 1).

**Figure 2:**
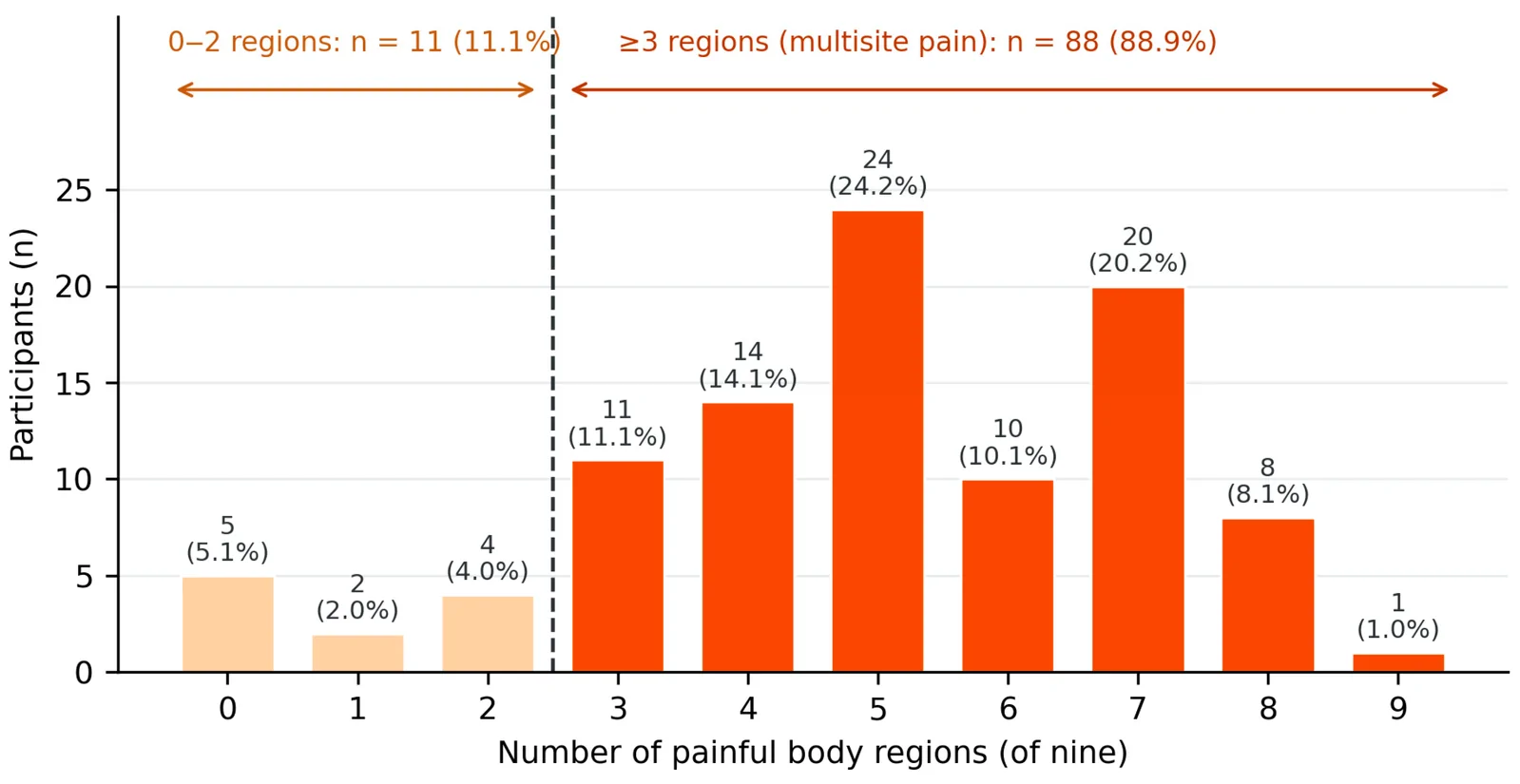
Distribution of the number of painful body regions (*n* = 99). Bars to the right of the dashed line represent participants classified as having multisite pain (three or more regions). Percentages are of the total sample.

The mean number of painful regions was 4.97 ± 2.09. Five participants (5.1%) reported no pain in any region, two (2.0%) reported pain in one region and four (4.0%) in two regions; the remaining 88 participants (88.9%) reported pain in three or more regions and were classified as having multisite pain (Table 3; Figure 2). Because the comparison group therefore includes participants with no pain at all, it is designated throughout as the 0–2 region group rather than the 1–2 region group.

Regarding symptom burden, 5.1% of participants reported never experiencing work-related pain, 31.3% occasionally, 47.5% often and 16.2% very often. Mean pain intensity was 3.51 ± 1.69 and mean QuickDASH score was 22.34 ± 12.03. Burnout subscale means were 22.78 ± 7.70 for emotional exhaustion, 12.12 ± 4.25 for depersonalisation and 13.66 ± 5.83 for reduced personal accomplishment (Table 4).

### 3.3. Comparisons by Pain Status

Across the nine body regions, 36 comparisons of burnout and QuickDASH scores by pain status were performed (Table 5). Five reached nominal significance before adjustment, but none survived either correction for multiplicity. Under the primary Holm correction the smallest adjusted *p*-value was 0.084, for the higher QuickDASH score among participants with upper back pain (25.17 ± 10.41 versus 16.67 ± 13.15, unadjusted *p* = 0.002); the Benjamini–Hochberg procedure, although less conservative, likewise left no comparison below 0.05. None of these regional comparisons therefore provides evidence of a region-specific association, and they are reported for completeness only.

**Figure 3:**
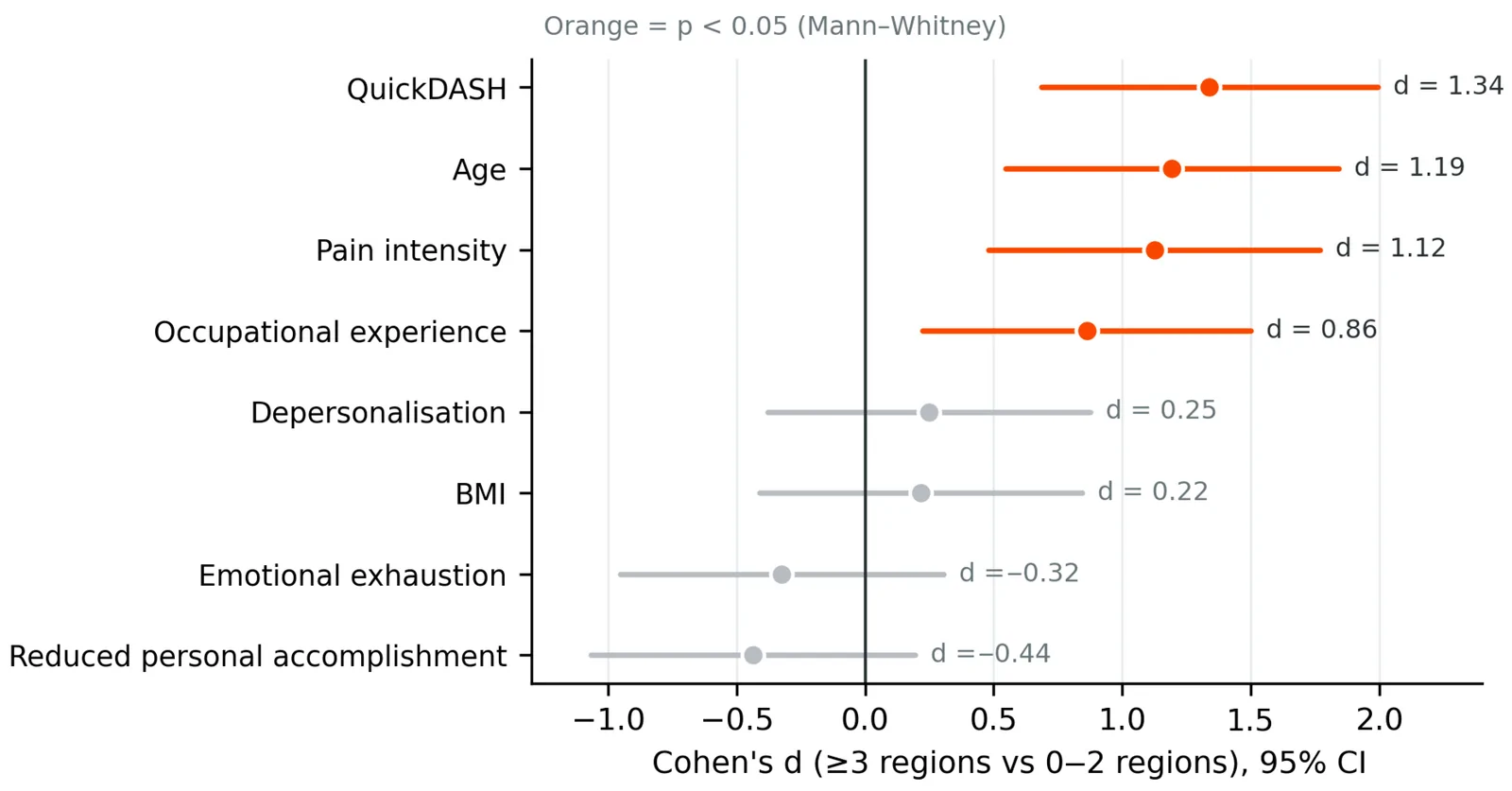
Standardised mean differences (Cohen’s d with 95% confidence intervals) between participants with three or more painful regions and those with 0–2 regions. Positive values indicate higher scores in the multisite pain group. Orange markers denote comparisons significant at *p* < 0.05 (Mann–Whitney U test); grey markers denote non-significant comparisons.

Participants with multisite pain were older (39.62 ± 7.02 versus 31.18 ± 7.53 years, *p* < 0.001, d = 1.19), more experienced (16.78 ± 7.15 versus 10.64 ± 6.96 years, *p* = 0.009, d = 0.86), and reported higher pain intensity (3.70 ± 1.53 versus 1.91 ± 2.07, *p* = 0.008, d = 1.12) and substantially higher QuickDASH scores (23.99 ± 10.92 versus 9.09 ± 12.74, *p* < 0.001, d = 1.34). No statistically significant difference was detected in BMI or in any burnout subscale (all *p* > 0.06) (Table 6; Figure 3). The observed standardised differences for the burnout subscales were small to moderate (d = −0.32 for emotional exhaustion, 0.25 for depersonalisation and −0.44 for reduced personal accomplishment) and all fell below the effect size this design could detect with 80% power (d ≈ 0.90), so these comparisons do not exclude differences in the magnitude observed.

### 3.4. Factors Associated with Multisite Pain

In univariable Firth penalised logistic regression, age (OR 1.18 per year, 95% CI 1.06–1.31, *p* = 0.003) and years of experience (OR 1.14 per year, 95% CI 1.02–1.27, *p* = 0.017) were associated with multisite pain. No statistically significant association was detected for BMI, daily working hours, weekly working days or any of the three burnout subscales (Table 7). Expressed per standard deviation, which makes the imprecision explicit, the burnout estimates were OR 0.73 (95% CI 0.39–1.39) for emotional exhaustion, 1.27 (0.69–2.35) for depersonalisation and 0.67 (0.38–1.18) for reduced personal accomplishment. Each of these intervals spans values corresponding to a substantial reduction and a substantial increase in odds, so the data are compatible with a range of true effects and do not support a conclusion that burnout is unrelated to multisite pain in this occupational group. For comparison, the corresponding estimate for age was OR 3.45 per standard deviation (95% CI 1.54–7.71).

In the multivariable model including age, BMI and emotional exhaustion, age remained the only variable independently associated with multisite pain (OR 1.17, 95% CI 1.05–1.30, *p* = 0.003); BMI (OR 1.08, *p* = 0.457) and emotional exhaustion (OR 0.98, *p* = 0.649) did not contribute.

**Figure 4:**
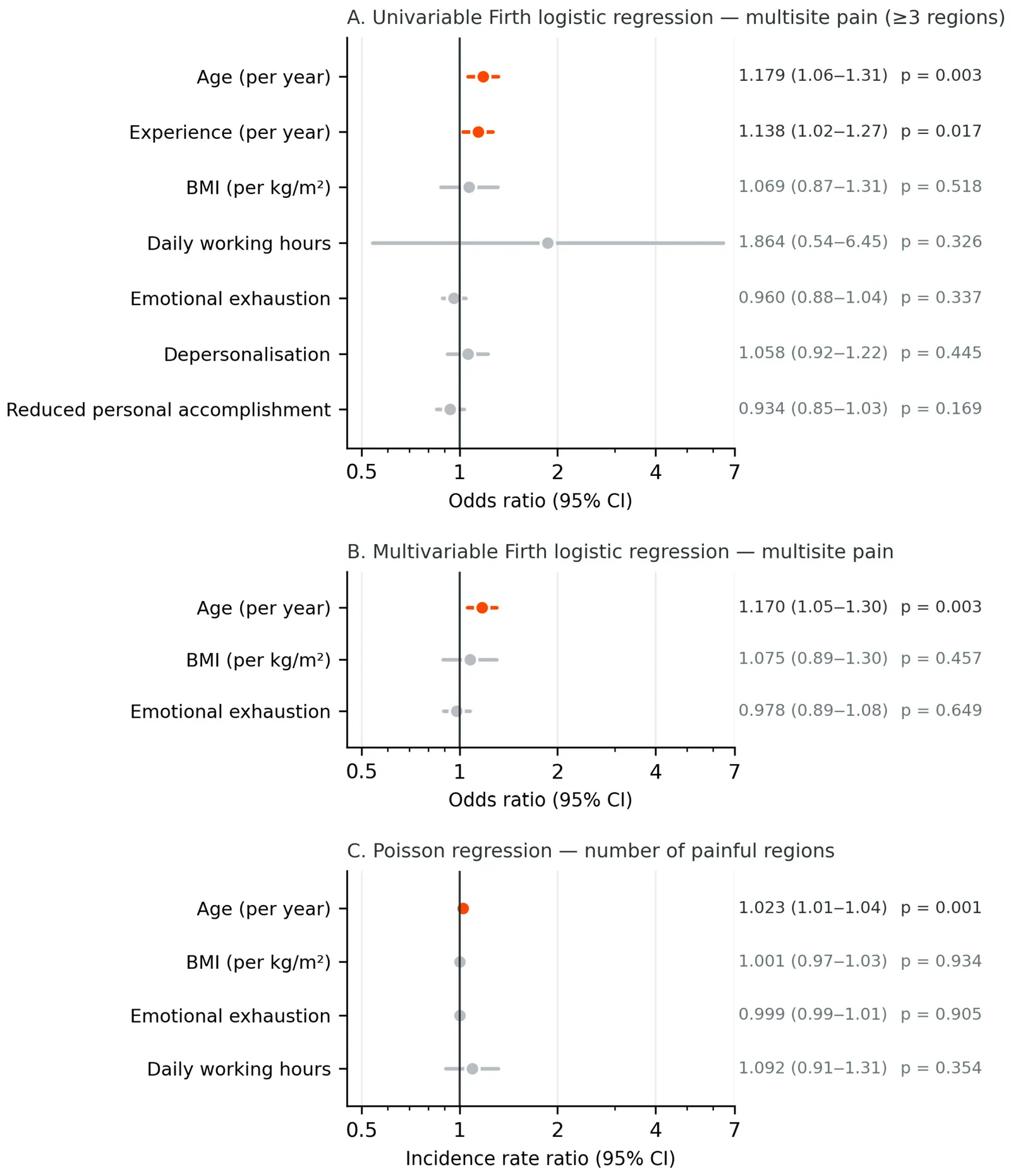
Forest plot of the regression models. (**A**) Univariable Firth penalised logistic regression for multisite pain; (**B**) multivariable Firth model; (**C**) Poisson regression for the number of painful regions. Markers show point estimates with 95% confidence intervals on a logarithmic scale; the vertical line marks the null value of 1.0. Orange denotes *p* < 0.05.

Modelling the number of painful regions directly as a count gave concordant results. In a Poisson model adjusted for BMI, emotional exhaustion and daily working hours, each additional year of age was associated with a 2.3% increase in the expected number of painful regions (IRR 1.023, 95% CI 1.010–1.036, *p* < 0.001), whereas no statistically significant association was detected for the other variables. The model fitted the data well and showed no evidence of over-dispersion: the deviance was 97.32 on 94 degrees of freedom (deviance/df = 1.04; goodness-of-fit *p* = 0.387) and the Pearson χ^2^ was 79.89 (Pearson/df = 0.85; goodness-of-fit *p* = 0.850). Both ratios lie close to or below unity, so a negative binomial specification was not required (Table 8; Figure 4).

### 3.5. Factors Associated with Upper-Extremity Disability

In the multiple linear regression model for QuickDASH including the number of painful regions, pain intensity, age, BMI and the three burnout subscales, only the number of painful regions contributed independently: each additional painful region was associated with a 1.58-point higher QuickDASH score (95% CI 0.30–2.85, *p* = 0.016). No statistically significant association was detected for pain intensity (*p* = 0.197), age, BMI or any burnout subscale (Table 9). Model assumptions were satisfied: all variance inflation factors were below 1.4; residuals did not depart from normality (Shapiro–Wilk *p* = 0.321; Jarque–Bera *p* = 0.378); there was no evidence of heteroscedasticity (Breusch–Pagan *p* = 0.129; White *p* = 0.271); the Durbin–Watson statistic was 1.77; and no observation was influential (maximum Cook’s distance 0.10, far below the conventional threshold of 1, and no externally studentised residual exceeded 3 in absolute value).

Despite these satisfactory diagnostics, the explanatory power of the model was low (R^2^ = 0.123; adjusted R^2^ = 0.056) and the overall model did not reach significance (F(7, 91) = 1.83, *p* = 0.092). The great majority of the variance in upper-extremity disability therefore remains unexplained by the variables measured here. The association with the number of painful regions is accordingly presented as an exploratory, hypothesis-generating finding rather than as evidence of a robust predictive relationship, and the model should not be used to predict QuickDASH scores in this population.

**Figure 5:**
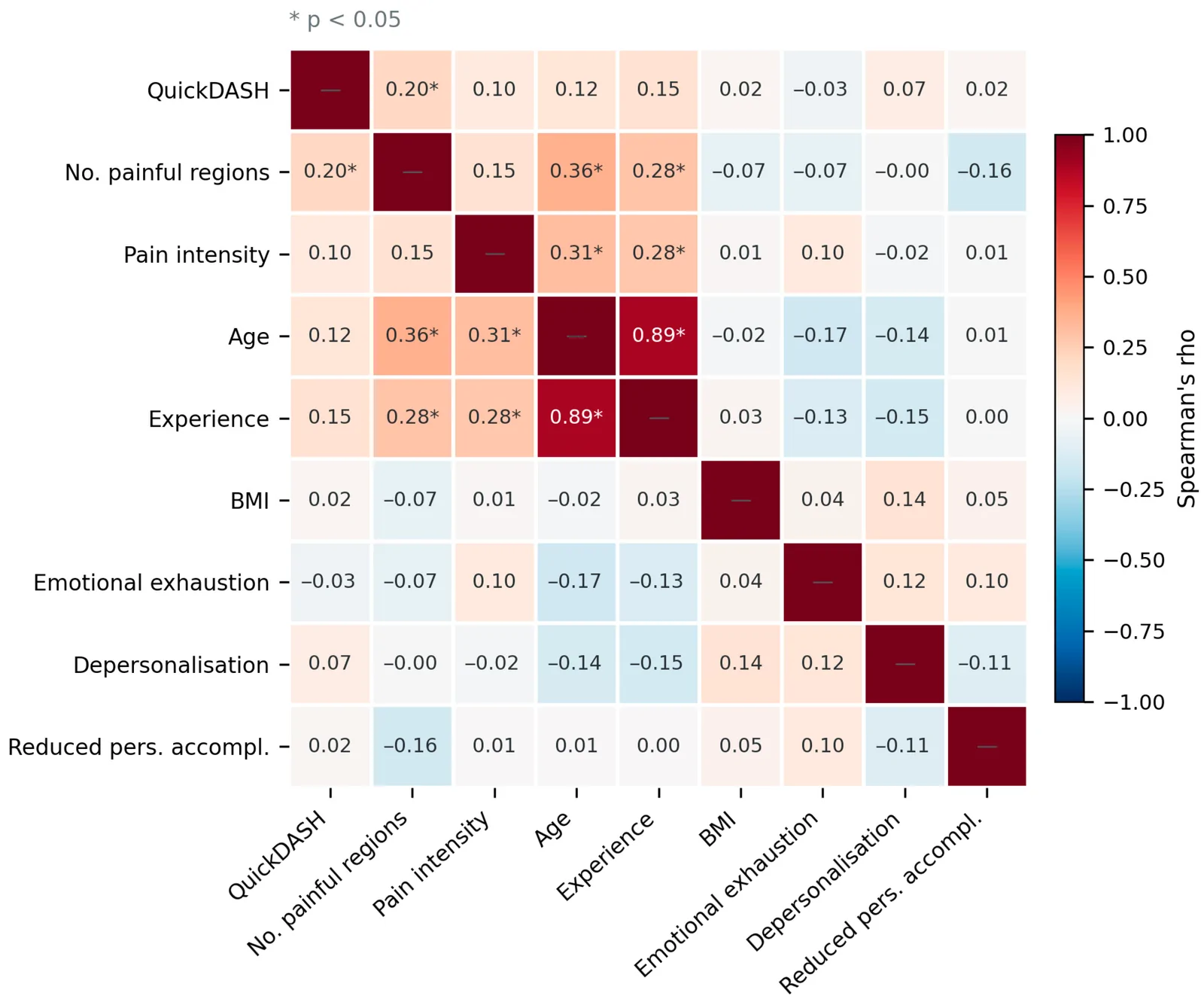
Spearman correlation matrix of pain, disability, demographic and burnout variables (*n* = 99). Cell values are correlation coefficients; asterisks denote *p* < 0.05. Warmer colours indicate positive and cooler colours negative correlations.

Spearman correlations were consistent with the regression findings: the number of painful regions correlated with age (ρ = 0.360, *p* < 0.001) and experience (ρ = 0.284, *p* = 0.004), and pain intensity correlated with age (ρ = 0.305, *p* = 0.002) and experience (ρ = 0.280, *p* = 0.005). No burnout subscale correlated significantly with any pain or disability measure (Figure 5).

## 4. Discussion

This study examined musculoskeletal symptom burden in a sample of male retail butchers in Istanbul, an occupational group that has attracted very little research attention in Türkiye. Three findings stand out. First, the burden was strikingly high: almost nine in ten participants reported pain in three or more body regions within the preceding seven days, with a mean of five painful regions per person. Second, age was the only variable consistently associated with this burden across two independent model specifications. Third, and contrary to our hypothesis, no statistically significant association was detected between any burnout dimension and multisite pain, the number of painful regions or upper-extremity disability. As set out below, this last result should be read as an absence of detected association in a sample with limited power for these comparisons, not as evidence that burnout is unrelated to musculoskeletal pain.

The prevalence figures deserve careful interpretation because of the recall window used. Most occupational studies of butchers and meat workers report twelve-month prevalence, and the scoping review literature places these estimates between roughly 10% and 88% depending on setting and region [9]. Our figures refer to the preceding seven days only and are therefore not directly comparable; studies that report both windows in slaughterhouse workers consistently find seven-day figures somewhat below twelve-month ones [6], which implies that the twelve-month burden in our sample would be higher still than the 88.9% observed here. The regional pattern we observed—upper back, shoulders and hand/wrist most affected—is broadly consistent with the exposure profile of the trade, in which sustained trunk flexion, repeated shoulder elevation and forceful gripping dominate [8], and with surveillance data from the meat sector showing shoulder lesions, tenosynovitis and carpal tunnel syndrome among the leading diagnoses [3]. It is also consistent with earlier work relating perceived bodily discomfort in slaughterhouse workers to individual and work-organisational factors [18].

The association with age merits comment. Each additional year of age was associated with roughly a 17% increase in the odds of multisite pain and a 2.2% increase in the expected number of painful regions. What this association represents cannot be determined from these data. Age and years in the trade were very strongly correlated in this sample (r = 0.887), and the variable we label age could equally be capturing cumulative occupational exposure, duration of employment, age-related changes in tissue tolerance and recovery capacity, or some combination of these. The two cannot be statistically separated here, and a cross-sectional design cannot establish which mechanism operates. One possible explanation is that symptom burden accumulates over the working life through repeated loading without adequate recovery; surveillance data from the meat sector likewise identify workers in the middle decades of working life as the most affected group [26], which is compatible with such an account. We present this as a hypothesis consistent with our data rather than as a demonstrated mechanism; longitudinal data, or a sample with greater independent variation in age and tenure, would be needed to test it. We also note that any such gradient is likely to be attenuated rather than exaggerated by selection: healthy-worker survivor effects would tend to remove the most severely affected individuals from the trade before they could be sampled.

The burnout result is the most notable negative finding, and its interpretation requires care. We did not detect a statistically significant association between any burnout dimension and any musculoskeletal outcome; this is not the same as showing that no association exists. The comparison group comprised only 11 participants, the design had 80% power to detect only large standardised differences (d ≈ 0.90), and the observed burnout effect sizes (d between −0.44 and 0.25) fell well below that threshold. The corresponding per-standard-deviation odds ratios spanned intervals ranging from substantial protection to substantial risk (for example 0.39 to 1.39 for emotional exhaustion). Firth penalisation addresses small-sample bias in the point estimates but cannot narrow these intervals. A moderate association between burnout and multisite pain in butchers is therefore entirely compatible with our data. This caveat is important because the literature is itself mixed. Longitudinal evidence in healthcare workers has linked burnout and stress to multisite pain, with sleep quality acting as a mediator [13], and cross-sectional work in white-collar employees has related musculoskeletal disorders to burnout and work engagement [15]; yet the occupations in which these associations are most consistently demonstrated are characterised by emotional labour and interpersonal demands rather than heavy physical loading [13,15,27]. A recent Turkish cross-sectional study of academicians likewise found no association between overall burnout and the number of painful regions, while job stress and impaired work–life balance were related to multisite pain [17]; in our own earlier work in male private security officers, emotional exhaustion was associated with multisite pain [12]. Two readings of our result are possible and cannot be distinguished here. Cumulative mechanical loading in this trade may dominate the symptom picture to an extent that leaves little variance for psychosocial factors to explain; alternatively, burnout as operationalised by an instrument developed for human-service occupations may simply not capture the psychosocial strains that matter for self-employed and small-shop tradespeople, whose principal stressors may relate to income insecurity, long hours and customer demand. Testing the second possibility would require instruments addressing job strain, job control, social support and effort–reward imbalance, constructs for which an association with musculoskeletal pain has been documented in systematic reviews [28], together with perceived stress, sleep quality and job satisfaction, none of which were administered here. This distinction governs how the present result may legitimately be used: our data speak to burnout as operationalised by the MBI, and to nothing else in the psychosocial domain. They do not support the inference that psychosocial factors are unimportant for musculoskeletal pain in butchers—a conclusion that would in any case run against the weight of the wider literature, in which perceived stress and sleep quality in particular have been linked to multisite pain [13,28].

The relationship between the number of painful regions and QuickDASH score was in the expected direction and is consistent with evidence that functional limitation increases approximately linearly with the number of pain sites [11], and with reports that musculoskeletal pain is associated with reduced work productivity [29]. Three qualifications apply. First, its practical magnitude is modest, approximately 1.6 QuickDASH points per additional region, so even the full observed range of region counts corresponds to a difference well within the measurement error of the instrument. Second, the model as a whole did not reach significance and explained only a small fraction of the variance, so the coefficient is an exploratory finding rather than a demonstrated predictive relationship. Third, and most importantly for interpretation, the two variables are not conceptually independent. The QuickDASH enquires about symptoms and limitations in the arm, shoulder and hand, and three of the nine Nordic regions counted in the outcome—shoulders, elbows and wrists/hands—lie in the same anatomical territory, so part of the observed association reflects shared item content rather than a relationship between distinct constructs. The association should accordingly be read as showing that participants with more widespread pain also report more upper-limb limitation, which is close to definitional, rather than as evidence that pain spread causes disability. It was for this same reason that the QuickDASH was excluded from the predictor set in the multisite pain models, a decision we consider necessary to avoid criterion contamination. A further observation bears on this point: age predicted the number of painful regions but showed no association with QuickDASH, either in the multivariable model or univariably (B = 0.25, *p* = 0.125; Spearman ρ = 0.121, *p* = 0.233), and the small age coefficient was fully attenuated once the number of painful regions was entered (B = 0.05, *p* = 0.749). The two outcomes are therefore not interchangeable: age appears to track how widely pain is distributed across the body rather than how much upper-limb function is lost, and what little age-related variation in QuickDASH exists is accounted for by the spread of pain itself.

Taken together, these findings highlight the potential importance of physical and ergonomic exposures in this occupational group and point to concrete targets: workstation and cutting-height adjustment, knife maintenance and grip design, task rotation, and scheduled micro-breaks during prolonged standing. Reviews of workplace interventions in poultry processing indicate that such measures can reduce symptom burden when they are matched to the specific task exposures of the setting [5], and randomised evidence in slaughterhouse workers supports structured workplace programmes for managing work-related musculoskeletal pain [30]. This emphasis should not be read as a recommendation to disregard psychosocial intervention. The present study measured only one psychosocial construct, and the absence of a detected association with that construct, in a sample with limited power, cannot be generalised to job strain, job control, social support, effort–reward imbalance, perceived stress, sleep quality, job satisfaction or income insecurity, none of which were assessed [28]. A balanced reading is that physical and ergonomic exposures are the better-evidenced target in this population at present, while the contribution of the wider psychosocial environment remains an open question requiring purpose-designed measurement.

What such a programme might contain can be specified a little more concretely, because the preventive and rehabilitative components with the strongest evidential support map onto the symptom distribution observed here. The first is therapeutic exercise matched to the loaded tissues: with the upper back, shoulders and wrists/hands most frequently affected, a programme addressing scapular and cervicothoracic endurance, rotator cuff strength, forearm and grip conditioning and thoracolumbar mobility would correspond to the regions actually at issue, and randomised evidence from other manually demanding occupations characterised by prolonged standing and forceful repetitive work indicates that adding a supervised exercise component to ergonomic training may be more effective than ergonomic training alone [31]. The second is structured health education with an explicit self-management element. Back school programmes, which combine group education on spinal loading, load handling and work pacing with supervised strengthening and flexibility training, have produced clinically relevant reductions in pain and disability in non-specific back pain in meta-analysis [19], and an eight-week back school-based programme improved pain, disability, kinesiophobia and the physical component of quality of life in a randomised controlled trial [20]; because pain in our sample was distributed across a mean of five regions, such a curriculum would need to be whole-body rather than confined to the lumbar spine. The third is multidisciplinary delivery, bringing physiotherapists and occupational health practitioners together with ergonomists and, where the workplace permits, employers’ representatives, since qualitative work with slaughterhouse workers indicates that acceptability and worker involvement largely determine whether these programmes are sustained [32]. Because symptom burden was higher among older and longer-serving butchers, measures introduced early in a working life may yield more benefit than remedial measures offered once symptoms are established; and because retail butchery is organised in small independent shops rather than large plants, delivery through trade chambers of the kind that gave access to the present sample may be more practical than employer-based occupational health provision. None of these components has been evaluated in retail butchers, so this is a programme design proposal arising from our descriptive findings and from evidence in adjacent occupations, not a set of recommendations validated in this trade.

### Limitations

Several limitations should be borne in mind. The design is cross-sectional, which precludes causal inference: exposure and outcome were recorded at the same moment, so no temporal ordering between them can be established, and age, experience and symptom burden may be related through pathways that this study cannot disentangle. The strong collinearity between age and tenure (r = 0.887) means, in addition, that neither can be isolated as the operative exposure. The seven-day recall window limits comparability with the largely twelve-month literature on this occupational group, and the twelve-month item, although present on the paper forms, was not available for analysis; this is the single most important measurement limitation of the study. A further consequence of the short recall window is that our estimates reflect symptom burden during a single three-month period in summer and early autumn; because workload in retail butchery varies across the year, seasonal variation in symptom reporting cannot be excluded, and the prevalence figures should not be read as an annual average. All outcomes were self-reported and no participant underwent clinical examination. Equally, no objective ergonomic assessment was carried out: no observational risk-assessment instrument such as RULA or REBA was applied, and posture, grip force, knife sharpness, load weight and repetition rate were neither measured nor systematically observed. Physical exposure is therefore represented in this study only by proxies—occupational experience and self-reported working hours—and misclassification of symptoms and reliance on perceived rather than measured workload cannot be excluded; for this reason we describe our outcomes throughout as musculoskeletal pain or symptoms rather than as diagnosed musculoskeletal disorders. Because the questionnaires were administered face to face by members of the research team rather than self-completed anonymously, residual social desirability and interviewer bias cannot be excluded, despite the scripted administration, private setting and explicit assurances described in Section 2.1.

Selection is a particular concern in a trade with a five-year minimum tenure criterion. Workers who developed severe symptoms or sustained disabling injuries in their first years are likely to have left butchery before they could be sampled, so the cohort studied here is one of survivors in the occupation. This healthy-worker survivor effect would be expected to understate the true burden of work-related musculoskeletal pain in the trade and to attenuate the observed gradients with age and tenure, and it also means that our estimates should not be read as describing everyone who has ever worked as a butcher. A second, smaller selection concern is non-response: although participation was high at 88.4%, the 13 butchers who declined did so without giving a reason and no information was recorded about them, so we cannot test whether they differed systematically from participants. Generalisability is further restricted in several directions. The sample comprised exclusively male butchers affiliated with a single chamber in one city; the findings cannot be extended to female workers, in whom both exposure patterns and the risk of work injury and disability may differ [33], to industrial meat-processing workers, whose tasks are subdivided and machine-paced, or to retail butchers in other regions of Türkiye or other countries. The eligibility window of 25 to 50 years excludes young entrants and older workers, precisely the groups at the ends of the age gradient we observed and therefore those in whom that gradient would be of greatest interest. Finally, no a priori sample size calculation was performed, and the marked imbalance between the two pain groups (11 versus 88) limits the precision of all comparisons involving the smaller group. Although Firth penalisation reduces small-sample bias, the resulting confidence intervals remain wide; this applies with particular force to the burnout analyses, which were powered to detect only large effects. The Poisson analysis of the region count, which does not depend on dichotomisation and showed satisfactory fit, provides some reassurance that the age association is not an artefact of the grouping.

Internal consistency coefficients could not be recomputed for the present sample because the analysis dataset contained scale and subscale totals rather than item-level responses; Cronbach’s alpha values for this cohort are therefore not reported, and the reliability evidence we cite is that of the Turkish validation studies of the respective instruments [21,23,24]. Finally, several potentially relevant covariates—smoking, comorbidities, physical activity outside work, shop-level organisational factors, perceived stress, sleep quality, job satisfaction, and objective measures of cutting-task exposure—were not recorded, and residual confounding by these factors cannot be excluded. Taken together, the single-city sampling frame, the exclusively male and relatively narrow age range, the imbalance between the two pain groups and the absence of objective exposure measurement mean that the findings are best read as a first description of symptom burden in this trade rather than as estimates that can be transferred to butchers in general.

## 5. Conclusions

Male retail butchers in this sample carried a very high seven-day burden of multisite musculoskeletal pain, with almost nine in ten reporting symptoms in three or more body regions and a mean of five painful regions per person. This burden was greater among older and longer-serving butchers, although the cross-sectional design and the strong collinearity between age and tenure do not allow these two explanations to be separated. No statistically significant association was detected between any burnout dimension and pain extent or upper-extremity disability; given the small comparison group and the correspondingly wide confidence intervals, this should be read as an absence of detected association rather than as evidence of no relationship, and it says nothing about psychosocial constructs that were not measured. To our knowledge this is the first study to characterise multisite musculoskeletal pain in Turkish retail butchers. The findings highlight the potential importance of cumulative physical and ergonomic exposures and suggest candidate targets for prevention in this group—workstation and tool design, task rotation, therapeutic exercise directed at the most affected regions, and early health education with a self-management component—while leaving open the contribution of psychosocial factors beyond burnout. These results are descriptive and hypothesis-generating: they derive from a single cross-sectional sample of male butchers in one city, without objective exposure assessment, and they establish associations rather than causes. Larger and preferably longitudinal studies, incorporating a broader set of psychosocial instruments and objective exposure assessment, are needed before they can be regarded as a basis for practice.

## Figures and Tables

**Table 1:** Sociodemographic and occupational characteristics of the participants (*n* = 99).

Variable	Mean ± SD	Min–Max	*n* (%)
Age (years)	38.69 ± 7.53	25–50	
Occupational experience (years)	16.10 ± 7.36	5–32	
Height (cm)	176.33 ± 7.36	163–190	
Weight (kg)	84.36 ± 11.70	60–115	
BMI (kg/m^2^)	27.09 ± 3.09	20.83–34.72	
Daily working hours	9.05 ± 0.50	8–10	
Weekly working days	5.91 ± 0.35	4–6	
Marital status: married			74 (74.7%)
Marital status: single			18 (18.2%)
Marital status: divorced			7 (7.1%)
Education: secondary school			22 (22.2%)
Education: high school			22 (22.2%)
Education: university			55 (55.6%)
Working 8 h/day			10 (10.1%)
Working 9 h/day			74 (74.7%)
Working 10 h/day			15 (15.2%)

**Table 2:** Seven-day prevalence of musculoskeletal pain by body region (*n* = 99).

Body Region	*n* with Pain	%
Upper back	66	66.7
Shoulders	63	63.6
Wrists/hands	59	59.6
Neck	58	58.6
Elbows	55	55.6
Low back	52	52.5
Hips/thighs	50	50.5
Knees	47	47.5
Ankles/feet	42	42.4

**Table 3:** Distribution of the number of painful regions (*n* = 99).

Number of Painful Regions	*n*	%
0	5	5.1
1	2	2.0
2	4	4.0
3	11	11.1
4	14	14.1
5	24	24.2
6	10	10.1
7	20	20.2
8	8	8.1
9	1	1.0

**Table 4:** Distribution of scale scores (*n* = 99).

Scale	Mean ± SD	Median	Min–Max	Skewness	Kurtosis	Shapiro–Wilk *p*
Emotional exhaustion (EE)	22.78 ± 7.70	24.00	8–35	−0.18	−1.03	<0.001
Depersonalisation (DP)	12.12 ± 4.25	12.00	3–20	−0.09	−0.97	0.002
Reduced personal accomplishment (PA)	13.66 ± 5.83	13.00	3–30	0.76	0.42	<0.001
QuickDASH	22.34 ± 12.03	25.00	0–43.18	−0.37	−0.86	<0.001
Pain intensity (VAS 0–10)	3.51 ± 1.69	3.00	0–8	0.14	−0.04	0.006
Number of painful regions (0–9)	4.97 ± 2.09	5.00	0–9	−0.53	−0.13	<0.001

**Table 5:** Burnout and QuickDASH scores by presence of pain in each body region, with multiplicity-adjusted *p*-values (36 comparisons).

Region	Scale	Pain Present	Pain Absent	Mann–Whitney *p*	Cohen d	Holm-Adjusted *p*	BH (FDR) *p*
Neck	Emotional exhaustion	21.98 ± 8.38	23.90 ± 6.56	0.197	−0.25	1.000	0.633
Neck	Depersonalisation	11.84 ± 3.90	12.51 ± 4.73	0.411	−0.16	1.000	0.769
Neck	Reduced personal accomplishment	13.66 ± 6.18	13.66 ± 5.36	0.800	−0.00	1.000	0.873
Neck	QuickDASH	24.69 ± 11.03	19.01 ± 12.71	0.037	0.48	1.000	0.292
Shoulders	Emotional exhaustion	22.40 ± 8.05	23.44 ± 7.12	0.486	−0.14	1.000	0.769
Shoulders	Depersonalisation	11.78 ± 4.05	12.72 ± 4.58	0.264	−0.22	1.000	0.731
Shoulders	Reduced personal accomplishment	13.29 ± 6.11	14.31 ± 5.32	0.364	−0.17	1.000	0.769
Shoulders	QuickDASH	23.66 ± 11.21	20.01 ± 13.17	0.211	0.31	1.000	0.633
Upper back	Emotional exhaustion	22.67 ± 8.24	23.00 ± 6.61	0.867	−0.04	1.000	0.892
Upper back	Depersonalisation	11.98 ± 4.15	12.39 ± 4.51	0.635	−0.10	1.000	0.816
Upper back	Reduced personal accomplishment	12.92 ± 5.87	15.12 ± 5.54	0.062	−0.38	1.000	0.374
Upper back	QuickDASH	25.17 ± 10.41	16.67 ± 13.15	0.002	0.75	0.084	0.084
Elbows	Emotional exhaustion	22.22 ± 8.04	23.48 ± 7.30	0.491	−0.16	1.000	0.769
Elbows	Depersonalisation	12.35 ± 4.22	11.84 ± 4.32	0.485	0.12	1.000	0.769
Elbows	Reduced personal accomplishment	12.85 ± 5.46	14.66 ± 6.17	0.134	−0.31	1.000	0.613
Elbows	QuickDASH	22.56 ± 11.09	22.06 ± 13.23	0.955	0.04	1.000	0.955
Wrists/hands	Emotional exhaustion	22.44 ± 8.17	23.27 ± 7.03	0.572	−0.11	1.000	0.809
Wrists/hands	Depersonalisation	12.22 ± 4.16	11.97 ± 4.43	0.736	0.06	1.000	0.872
Wrists/hands	Reduced personal accomplishment	12.54 ± 5.78	15.30 ± 5.56	0.011	−0.48	0.390	0.200
Wrists/hands	QuickDASH	23.96 ± 11.07	19.94 ± 13.09	0.161	0.34	1.000	0.633
Low back	Emotional exhaustion	24.00 ± 8.20	21.43 ± 6.96	0.136	0.34	1.000	0.613
Low back	Depersonalisation	12.25 ± 4.18	11.98 ± 4.37	0.751	0.06	1.000	0.872
Low back	Reduced personal accomplishment	13.35 ± 6.11	14.00 ± 5.54	0.459	−0.11	1.000	0.769
Low back	QuickDASH	23.08 ± 12.06	21.52 ± 12.06	0.540	0.13	1.000	0.809
Hips/thighs	Emotional exhaustion	22.00 ± 8.28	23.57 ± 7.06	0.386	−0.20	1.000	0.769
Hips/thighs	Depersonalisation	12.60 ± 4.02	11.63 ± 4.47	0.191	0.23	1.000	0.633
Hips/thighs	Reduced personal accomplishment	13.38 ± 6.37	13.94 ± 5.27	0.400	−0.10	1.000	0.769
Hips/thighs	QuickDASH	25.23 ± 9.50	19.39 ± 13.62	0.041	0.50	1.000	0.292
Knees	Emotional exhaustion	22.89 ± 8.74	22.67 ± 6.72	0.787	0.03	1.000	0.873
Knees	Depersonalisation	11.70 ± 4.03	12.50 ± 4.45	0.425	−0.19	1.000	0.769
Knees	Reduced personal accomplishment	13.21 ± 6.26	14.06 ± 5.43	0.361	−0.14	1.000	0.769
Knees	QuickDASH	23.50 ± 10.27	21.28 ± 13.44	0.602	0.18	1.000	0.809
Ankles/feet	Emotional exhaustion	22.38 ± 7.91	23.07 ± 7.61	0.607	−0.09	1.000	0.809
Ankles/feet	Depersonalisation	12.33 ± 4.22	11.96 ± 4.31	0.708	0.09	1.000	0.872
Ankles/feet	Reduced personal accomplishment	15.14 ± 6.13	12.56 ± 5.38	0.022	0.45	0.764	0.270
Ankles/feet	QuickDASH	22.56 ± 12.02	22.17 ± 12.14	0.862	0.03	1.000	0.892

Mann–Whitney U test reported as the primary test. Holm and Benjamini–Hochberg corrections were computed across all 36 comparisons. No comparison remained significant after adjustment.

**Table 6:** Comparison of participants with 0–2 versus three or more painful regions.

Variable	0–2 Regions (*n* = 11)	3+ Regions (*n* = 88)	Mann–Whitney *p*	Welch *t*-Test *p*	Cohen d
Age	31.18 ± 7.53	39.62 ± 7.02	<0.001	0.004	1.19
Occupational experience	10.64 ± 6.96	16.78 ± 7.15	0.009	0.017	0.86
BMI	26.50 ± 3.50	27.17 ± 3.05	0.432	0.556	0.22
Emotional exhaustion	25.00 ± 5.00	22.50 ± 7.96	0.310	0.166	−0.32
Depersonalisation	11.18 ± 5.13	12.24 ± 4.15	0.410	0.524	0.25
Reduced personal accomplishment	15.91 ± 3.56	13.38 ± 6.00	0.064	0.058	−0.44
QuickDASH	9.09 ± 12.74	23.99 ± 10.92	<0.001	0.003	1.34
Pain intensity	1.91 ± 2.07	3.70 ± 1.53	0.008	0.017	1.12

**Table 7:** Firth penalised logistic regression for multisite pain (three or more regions).

Model	Variable	OR	95% CI	*p*
Univariable (Firth)	Age (years)	1.18	1.06–1.31	0.003
Univariable (Firth)	Occupational experience (years)	1.14	1.02–1.27	0.017
Univariable (Firth)	BMI	1.07	0.87–1.31	0.518
Univariable (Firth)	Daily working hours	1.86	0.54–6.45	0.326
Univariable (Firth)	Weekly working days	1.36	0.31–6.03	0.683
Univariable (Firth)	Emotional exhaustion	0.96	0.88–1.04	0.337
Univariable (Firth)	Depersonalisation	1.06	0.92–1.22	0.445
Univariable (Firth)	Reduced personal accomplishment	0.93	0.85–1.03	0.169
Multivariable (Firth)	Age (years)	1.17	1.05–1.30	0.003
Multivariable (Firth)	BMI	1.07	0.89–1.30	0.457
Multivariable (Firth)	Emotional exhaustion	0.98	0.89–1.08	0.649

**Table 8:** Poisson regression for the number of painful regions.

Variable	IRR	95% CI	*p*
Age (years)	1.023	1.010–1.036	<0.001
BMI	1.001	0.972–1.032	0.934
Emotional exhaustion	0.999	0.988–1.011	0.905
Daily working hours	1.092	0.907–1.314	0.354

**Table 9:** Multiple linear regression for QuickDASH score.

**Variable**	**B**	**SE**	**Beta**	**95% CI**	***p***	**VIF**
Number of painful regions	1.58	0.64	0.274	0.30–2.85	0.016	1.29
Pain intensity	0.98	0.76	0.138	−0.52–2.48	0.197	1.17
Age	−0.01	0.18	−0.004	−0.37–0.35	0.971	1.34
BMI	0.23	0.39	0.059	−0.54–1.00	0.555	1.03
Emotional exhaustion	−0.10	0.16	−0.064	−0.41–0.21	0.530	1.07
Depersonalisation	0.15	0.29	0.053	−0.42–0.72	0.604	1.06
Reduced personal accomplishment	0.06	0.21	0.031	−0.35–0.48	0.757	1.05

R^2^ = 0.123|Adjusted R^2^ = 0.056|F(7, 91) = 1.83, *p* = 0.092.

## Data Availability

The original contributions presented in this study are included in the article. Further inquiries can be directed to the corresponding author.
